# GLP-1 Receptor Agonists Across the Coronary Disease Continuum in Type 2 Diabetes: A Systematic Review of Ischemic Events, Revascularization, and Stent Healing

**DOI:** 10.7759/cureus.117500

**Published:** 2026-10-05

**Authors:** Batool I Abuzenah, Hebah Abuzenah, Mohammad Abuzenah

**Affiliations:** 1 School of Pharmacy, University of Jordan, Amman, JOR; 2 School of Pharmacy, Al-Ahliyya Amman University, Amman, JOR; 3 Internal Medicine, Northern Care Alliance NHS Foundation Trust, Salford, GBR

**Keywords:** coronary revascularization, glp-1 receptor agonists, myocardial infarction, percutaneous coronary intervention, type 2 diabetes mellitus

## Abstract

Type 2 diabetes is associated with accelerated coronary atherosclerosis, recurrent ischemic events, and impaired vascular healing after percutaneous coronary intervention (PCI); however, the effects of glucagon-like peptide-1 (GLP-1) receptor agonists across these distinct coronary outcomes remain incompletely characterized. This systematic review synthesized randomized evidence on myocardial infarction (MI), acute coronary syndrome (ACS), coronary revascularization, post-PCI healing, and mechanistic markers of coronary disease. MEDLINE via PubMed, Scopus, Web of Science Core Collection, and the Cochrane Central Register of Controlled Trials (CENTRAL) were initially searched for eligible reports published from January 1, 2020, through December 31, 2025. All four database searches were updated on August 31, 2026, extending publication eligibility through that date; no additional eligible reports were identified. The review was conducted in accordance with the Preferred Reporting Items for Systematic Reviews and Meta-Analyses (PRISMA) 2020 statement. Seven eligible reports derived from seven parent randomized trials, involving 37,033 participants, were included. Two presented primary findings from independent randomized trials, three were prespecified analyses of parent randomized trials, and two were post hoc analyses. Owing to substantial clinical and outcome heterogeneity, the findings were synthesized narratively. Albiglutide reduced first and recurrent MI, including type 1 and type 2 events, whereas higher-dose efpeglenatide yielded more favorable cardiovascular effect estimates than the lower dose. Across separate reports, semaglutide favorably modified epicardial adipose tissue, a surrogate imaging measure, and reduced the composite of cardiovascular death, MI, or stroke in patients with diabetic kidney disease. Liraglutide was associated with lower risks of broader cardiovascular composites compared with the other glucose-lowering therapies combined, although individual MI and revascularization outcomes were not significantly reduced. Exenatide did not significantly reduce the composite of hospitalization for ACS or coronary revascularization, and the only PCI-specific trial demonstrated no significant improvement in early stent coverage, neointimal thickness, or in-stent obstruction. Of the seven included reports, one was judged to have a low risk of bias, whereas six raised some concerns, primarily because of post hoc analyses, open-label treatment, incomplete methodological reporting, or limited imaging follow-up. Across the included reports, findings were more favorable for MI and broader cardiovascular events than for coronary revascularization or post-stent healing. However, the small number of heterogeneous reports, reliance on several secondary or post hoc analyses, and absence of quantitative meta-analysis limit firm conclusions and do not establish a definitive class-wide coronary effect. Potential differences by agent, dose, and patient phenotype require further investigation, while treatment-initiation timing remains largely untested.

## Introduction and background

Type 2 diabetes mellitus (T2DM) is a major determinant of coronary morbidity and mortality through accelerated atherosclerosis, endothelial dysfunction, vascular inflammation, platelet activation, and adverse arterial remodeling. Patients with T2DM are more likely to develop diffuse and multivessel coronary disease, experience myocardial infarction (MI), and require percutaneous or surgical revascularization [1]. This risk persists after coronary blood flow has been restored. Following percutaneous coronary intervention (PCI), impaired endothelial recovery, increased neointimal proliferation, and persistent metabolic and inflammatory activity may contribute to restenosis, stent thrombosis, target-lesion revascularization, and disease progression in untreated coronary segments. Coronary risk in T2DM therefore spans a continuum from vulnerable native-vessel plaques to the vascular response after mechanical intervention [2].

Glucagon-like peptide-1 receptor agonists (GLP-1 RAs) have become central to the contemporary management of T2DM because selected agents reduce major adverse cardiovascular events (MACE) while improving glycemic control and promoting weight loss. Their cardiovascular effects may involve several interacting pathways, including reductions in adiposity, blood pressure, systemic inflammation, oxidative stress, and atherogenic metabolic burden [3]. Experimental and imaging evidence has also suggested more direct vascular effects involving endothelial function, plaque composition, epicardial adipose tissue, platelet activity, and arterial inflammation. These mechanisms provide a plausible basis for coronary protection, but their relative contributions to the observed clinical benefits remain uncertain and may vary according to the agent, dose, treatment duration, and patient phenotype [4].

Importantly, a reduction in MI does not necessarily correspond to reduced coronary revascularization or improved healing after stent implantation. MI may be prevented through modification of plaque vulnerability, thrombosis, or myocardial oxygen balance, whereas revascularization is also influenced by fixed anatomic obstruction, symptom burden, ischemia testing, clinical decision-making, health care access, and regional practice patterns [5,6]. Post-PCI healing represents a distinct process governed by endothelial recovery, neointimal growth, stent characteristics, antiplatelet therapy, and local vascular injury. Although large cardiovascular outcome trials provide substantial evidence on ischemic events, comparatively little randomized evidence has directly examined restenosis, target-lesion revascularization, stent healing, native-vessel disease progression, or the influence of GLP-1 RA initiation timing relative to PCI [7].

Accordingly, this systematic review aimed to synthesize randomized evidence on the effects of GLP-1 RAs on coronary outcomes in adults with T2DM, including MI, acute coronary syndrome, coronary revascularization, post-PCI vascular healing, restenosis, and mechanistic markers of coronary disease progression. It also sought to determine whether the available evidence supports a consistent coronary effect or an outcome-specific pattern of benefit and whether the findings vary according to the agent, dose, cardiovascular phenotype, and treatment duration. Particular attention was given to treatment-initiation timing as a clinically relevant but insufficiently studied modifier of coronary response.

## Review

Materials and methods

Study Design and Reporting Framework

This systematic review was conducted in accordance with the Preferred Reporting Items for Systematic Reviews and Meta-Analyses (PRISMA) 2020 statement [8]. An internal review protocol was developed before study selection but was neither prospectively registered in PROSPERO nor made publicly available. The protocol specified the eligibility criteria, information sources and search approach, study-selection and data-extraction procedures, risk-of-bias assessment, and planned evidence synthesis before screening commenced. These prespecified methods were intended to promote consistency and limit post-hoc decision-making. However, the absence of a publicly accessible, dated protocol limits independent verification of this prespecification and assessment of any subsequent methodological deviations. We therefore acknowledge nonregistration and the lack of public protocol availability as limitations of review transparency. The review focused on randomized evidence regarding the effects of GLP-1 RAs on clinical coronary outcomes and coronary vascular responses in adults with T2DM.

Research Question and PICO Framework

The review question was structured according to the Population, Intervention, Comparator, and Outcome (PICO) framework. The population comprised adults aged 18 years or older with T2DM, irrespective of baseline cardiovascular risk, established atherosclerotic cardiovascular disease (ASCVD), chronic kidney disease (CKD), previous myocardial infarction (MI), or percutaneous coronary intervention (PCI) status. The intervention consisted of treatment with a GLP-1 RA, including semaglutide, liraglutide, exenatide, albiglutide, or efpeglenatide. Eligible comparators included placebo, standard care, or an active glucose-lowering therapy. Outcomes included MI, acute coronary syndrome (ACS), coronary revascularization, target-lesion or target-vessel revascularization, in-stent restenosis, stent thrombosis, endothelial or stent-strut coverage, neointimal response, native-vessel atherosclerotic progression, coronary plaque characteristics, and epicardial adipose tissue. Cardiovascular composite outcomes were eligible if they explicitly included MI, ACS, or coronary revascularization.

Information Sources and Search Period

MEDLINE via PubMed, Scopus, Web of Science Core Collection, and the Cochrane Central Register of Controlled Trials (CENTRAL) were initially searched for eligible reports published from January 1, 2020, through December 31, 2025. Searches of all four databases were subsequently updated on August 31, 2026, extending publication eligibility through that date. No additional reports meeting the eligibility criteria were identified in the update. This search period was selected to capture contemporary randomized evidence involving clinically relevant GLP-1 RAs, cardiovascular outcome trials, modern drug-eluting stents, and current coronary imaging techniques. The reference lists of included reports and relevant reviews were also examined to identify potentially eligible reports not retrieved through the electronic database searches.

Clinical trial registries and other dedicated sources of unpublished or ongoing studies were not searched. The search approach focused on published reports providing quantitative clinical or mechanistic coronary outcomes suitable for synthesis. However, this approach may have missed unpublished results or eligible trials with selectively available outcome data. Registry searching could also have identified ongoing studies and completed trials without published findings; its omission is therefore acknowledged as a limitation.

Search Strategy

The search combined controlled vocabulary, where available, with free-text terms related to T2DM, GLP-1 RAs, coronary disease, PCI, and randomized evidence. Search terms included “type 2 diabetes,” “T2DM,” “GLP-1 receptor agonist,” “semaglutide,” “liraglutide,” “exenatide,” “albiglutide,” “efpeglenatide,” “myocardial infarction,” “acute coronary syndrome,” “coronary revascularization,” “percutaneous coronary intervention,” “stent,” “endothelialization,” “in-stent restenosis,” “target-lesion revascularization,” “coronary plaque,” “atherosclerosis progression,” and “epicardial adipose tissue.” These terms were combined with methodological terms such as “randomized,” “placebo,” “clinical trial,” “secondary analysis,” and “post hoc analysis.” Representative combinations included “type 2 diabetes” AND “GLP-1 receptor agonist” AND “myocardial infarction”; “type 2 diabetes” AND “GLP-1 receptor agonist” AND “coronary revascularization”; and “GLP-1 receptor agonist” AND “PCI” AND (“stent healing” OR “restenosis”). The Boolean operator AND connected the principal concepts, whereas OR combined synonyms, individual agents, and related outcomes. Search syntax was adapted to the indexing requirements of each database. Appendix A summarizes the search terms, Boolean structure, database-specific search fields, publication eligibility periods, and final search dates.

Eligibility Criteria

Eligible reports comprised primary publications of randomized controlled trials and prespecified, secondary, subgroup, dose-response, or post hoc analyses derived from parent randomized trials. Secondary and post hoc reports were eligible only when participants remained compared according to their original randomized treatment allocation and when the reports provided additional coronary outcomes not adequately presented in the primary trial publication. This approach permitted the inclusion of reports from direct PCI and imaging trials, mechanistic trials evaluating coronary adipose tissue or vascular response, comparative-effectiveness trials in lower-risk diabetes populations, and cardiovascular outcome trials involving participants with established ASCVD or CKD. Reports were required to present at least one eligible clinical or mechanistic coronary outcome with sufficient quantitative information for interpretation. Reports presenting broader cardiovascular composites were eligible when MI, ACS, or coronary revascularization formed an explicit component of the endpoint.

Reports were excluded if they described observational, nonrandomized, single-arm, preclinical, animal, or in vitro investigations; enrolled participants without T2DM unless separate randomized data for the eligible population were available; evaluated an intervention without a GLP-1 RA component; or presented only glycemic, weight, renal, or general cardiovascular outcomes without an identifiable coronary component. Reviews, editorials, protocols, case reports, conference abstracts without adequate outcome data, and duplicate reports that did not contribute additional relevant findings were also excluded.

Study Selection and Data Extraction

All retrieved records were consolidated, and duplicates were removed before screening. Two reviewers independently screened the titles and abstracts and subsequently assessed the full texts of potentially eligible reports. Disagreements were resolved through discussion and consensus, with consultation of a third reviewer when necessary. Reasons for exclusion during full-text assessment were documented for inclusion in the PRISMA flow diagram. Using a standardized extraction form, two reviewers independently extracted data on publication type, parent trial, study design, sample size, population characteristics, cardiovascular risk profile, PCI status, intervention, dose, comparator, treatment duration, follow-up, outcome definitions, event numbers, effect estimates, confidence intervals, and P values. When multiple publications originated from the same parent trial, each publication was considered a distinct eligible report only if it contributed a separate coronary outcome relevant to the review.

Risk of Bias Assessment

Two reviewers independently evaluated the risk of bias using the Cochrane Risk of Bias 2 (RoB 2) tool for randomized trials [9]. The assessment addressed bias arising from the randomization process, deviations from intended interventions, missing outcome data, outcome measurement, and selection of the reported result. Each result contributing to the review was judged as having a low risk of bias, some concerns, or a high risk of bias. Secondary and post hoc analyses were assessed within the randomized-trial framework, with particular attention to outcome prespecification, multiplicity, exploratory comparisons, and selective reporting. Disagreements were resolved through consensus or consultation with a third reviewer.

Data Synthesis

Because the included reports drew on parent trials that differed substantially in clinical population, GLP-1 RA, dose, comparator, follow-up duration, and outcome construct, a structured narrative synthesis was undertaken. Evidence was organized into clinical ischemic outcomes, coronary revascularization outcomes, post-PCI vascular-healing measures, and mechanistic markers of coronary substrate modification. Hazard ratios, confidence intervals, event rates, and P values were reported as presented in the original reports. A pooled meta-analysis was not performed because MI, revascularization, epicardial adipose tissue, and optical coherence tomography (OCT)-derived stent-healing measures represent clinically and biologically distinct outcomes, and combining them would have produced a summary estimate of uncertain interpretability.

Results

Study Selection Process

The database search identified 641 records, of which 36 duplicates were removed before screening. The titles and abstracts of the remaining 605 records were screened, and 489 were excluded. Full-text retrieval was sought for 116 reports; however, 11 could not be retrieved, leaving 105 reports for eligibility assessment. Of these, 98 were excluded because of an ineligible study design, population, intervention, or outcome; insufficiently reported data; or duplication without additional relevant findings. Ultimately, seven eligible reports, each derived from a distinct parent randomized trial, were included in the systematic review. The complete study selection process and reasons for exclusion during full-text assessment are presented in Figure 1.

**Figure 1:**
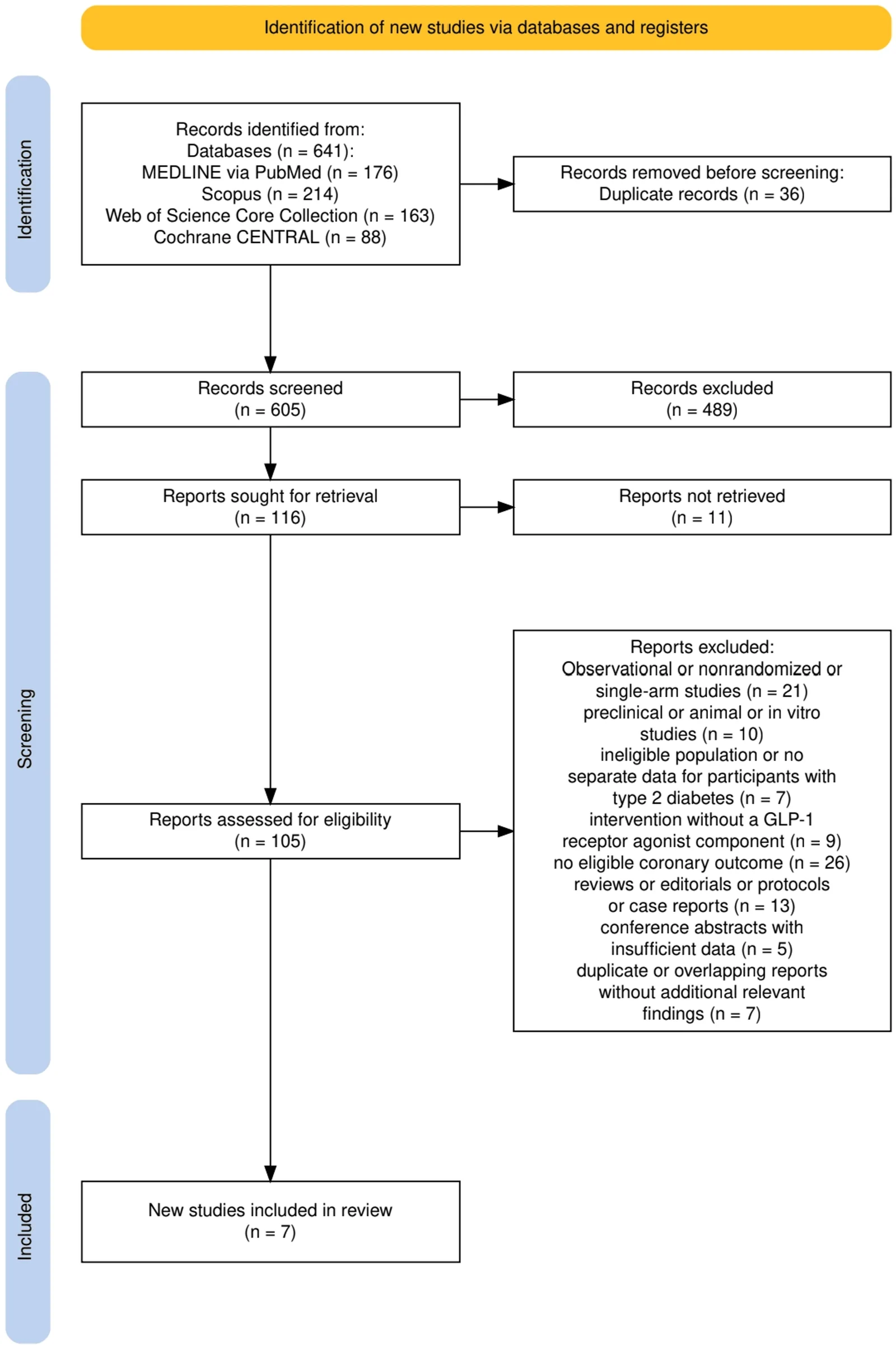
PRISMA flow diagram representing the study selection process. PRISMA, Preferred Reporting Items for Systematic Reviews and Meta-Analyses.

Characteristics of the Selected Studies

Seven eligible reports, each derived from a distinct parent randomized trial and collectively involving 37,033 participants, were included. Two reports presented primary findings from independent randomized trials, three were prespecified analyses of parent randomized trials, and two were post hoc analyses. Across these reports, semaglutide, liraglutide, efpeglenatide, exenatide, and albiglutide were compared with placebo, standard care, or active glucose-lowering therapies. The parent-trial populations ranged from adults with relatively recent T2DM and low cardiovascular risk to those with established ASCVD, CKD, or recent PCI. Follow-up ranged from 12 weeks to five years, and the reported outcomes included MI, ACS, coronary revascularization, cardiovascular composites, epicardial adipose tissue, and OCT-derived stent-healing measures. Detailed characteristics of the included reports and their parent randomized trials are presented in Table 1.

**Table 1:** Characteristics, coronary outcomes, statistical findings, and clinical interpretation of the included reports and their parent randomized trials. ACS, acute coronary syndrome; AMPLITUDE-O, Effect of Efpeglenatide on Cardiovascular Outcomes; ASCVD, atherosclerotic cardiovascular disease; CI, confidence interval; CKD, chronic kidney disease; CT, computed tomography; EAT, epicardial adipose tissue; eGFR, estimated glomerular filtration rate; EXSCEL, Exenatide Study of Cardiovascular Event Lowering; FLOW, Evaluate Renal Function With Semaglutide Once Weekly; GRADE, Glycemia Reduction Approaches in Type 2 Diabetes: A Comparative Effectiveness Study; HR, hazard ratio; HU, Hounsfield units; IQR, interquartile range; KDIGO, Kidney Disease: Improving Global Outcomes; MACE, major adverse cardiovascular events; MACE-5 and MACE-6, five- and six-component major adverse cardiovascular event composites, respectively; MI, myocardial infarction; NSTEMI, non-ST-segment elevation myocardial infarction; OCT, optical coherence tomography; PCI, percutaneous coronary intervention; RCT, randomized controlled trial; REBUILD, Effects of Exenatide on Coronary Stent Endothelialization in Subjects With Type 2 Diabetes study; STEMI, ST-segment elevation myocardial infarction; STOP, Semaglutide Treatment Effect on Coronary Atherosclerosis Progression; T2D, type 2 diabetes.

Report/parent trial	Design and population	Intervention vs. comparator	Treatment and follow-up	Coronary outcome(s)	Key statistical findings	Clinical interpretation
Manubolu et al., 2024, STOP [10]	Primary report of the single-center, double-blind STOP randomized trial; 140 adults with T2D	Semaglutide vs placebo	Semaglutide titrated from 0.25 to 1 mg once weekly; 52 weeks	CT-measured epicardial adipose tissue (EAT) volume and density as markers related to coronary atherosclerotic activity	EAT volume decreased with semaglutide but increased with placebo: −10.7 ± 24.6 vs +6.4 ± 22.8 cm³; P=0.001. EAT density increased: +4.1 ± 9.6 vs −1.6 ± 9.0 HU; P=0.001.	Semaglutide favorably modified EAT, suggesting a potential coronary anti-inflammatory or plaque-modifying effect. However, EAT is a surrogate marker, and clinical coronary events or stent outcomes were not assessed.
Green et al., 2024, GRADE [11]	Prespecified cardiovascular-outcome analysis of the parent multicenter, open-label GRADE randomized trial; 5,047 adults with relatively recent T2D, largely without established cardiovascular disease	Liraglutide vs insulin glargine, glimepiride, or sitagliptin, all added to metformin	Liraglutide up to 1.8 mg daily; median follow-up, five years	MACE composites incorporating MI, unstable angina, and coronary revascularization; individual cardiovascular events	No significant differences across all four randomized groups. Liraglutide vs the other treatments combined reduced MACE-5: adjusted HR 0.70 (95% CI 0.54–0.91; P=0.021) and MACE-6: adjusted HR 0.70 (95% CI 0.55–0.90; P=0.021). Individual MI and revascularization outcomes were not significantly reduced.	Liraglutide may reduce the broader cardiovascular-event burden in relatively low-risk T2D, but the report does not establish an independent benefit for MI or coronary revascularization.
Gerstein et al., 2023, AMPLITUDE-O [12]	Prespecified dose-response analysis of the parent multicenter, double-blind, placebo-controlled AMPLITUDE-O randomized trial; 4,076 adults with T2D and high cardiovascular risk	Efpeglenatide 6 mg or 4 mg weekly vs placebo	Subcutaneous treatment once weekly; median follow-up, 1.8 years	MACE and an expanded composite incorporating MACE, coronary revascularization, or hospitalization for unstable angina	MACE occurred in 6.2%, 7.7%, and 9.2% of participants receiving efpeglenatide 6 mg, efpeglenatide 4 mg, and placebo, respectively. Compared with placebo, the HR was 0.65 (95% CI 0.50–0.86; P=0.0027) for 6 mg and 0.82 (95% CI 0.63–1.06; P=0.14) for 4 mg. For the expanded cardiovascular composite, the corresponding HRs were 0.73 (P=0.011) and 0.85 (P=0.17). Although the individual 4-mg comparisons were not statistically significant, tests for dose-response trend across the placebo, 4-mg, and 6-mg groups were significant for all primary and secondary outcomes (all P for trend ≤0.018).	The graded estimates and significant trend tests suggest a possible dose-response relationship, with more favorable cardiovascular effects at 6 mg. However, the individual 4-mg comparisons were nonsignificant, and the independent contribution of coronary revascularization to the expanded composite could not be determined.
Mahaffey et al., 2025, FLOW [13]	Prespecified cardiovascular analysis of the parent multicenter, double-blind, placebo-controlled FLOW randomized trial; 3,533 adults with T2D and CKD, predominantly at high or very high kidney risk	Semaglutide vs placebo, alongside standard care	Semaglutide 1 mg subcutaneously once weekly; median follow-up, 3.4 years	Composite of cardiovascular death, nonfatal MI, or nonfatal stroke, examined across CKD-severity categories	Semaglutide reduced cardiovascular death, MI, or stroke: HR 0.82 (95% CI 0.68–0.98; P=0.03). Effects were consistent across eGFR, albuminuria, and KDIGO-risk categories, with all relevant interaction P>0.13. All-cause mortality: HR 0.80 (95% CI 0.67–0.95; P=0.01).	Semaglutide reduced major cardiovascular events across CKD-severity groups. The benefit applies to a composite outcome and does not independently establish an effect on MI, PCI, or revascularization.
Santos-Pardo et al., 2023, REBUILD [14]	Primary report of the multicenter, open-label, assessor-blinded REBUILD randomized trial; 38 adults with T2D undergoing PCI with a modern drug-eluting stent	Exenatide plus standard treatment vs standard treatment alone	Extended-release exenatide 2 mg once weekly; repeat angiography and OCT after 12 weeks	Stent-strut coverage, neointimal thickness, maximal in-stent obstruction, neoatherosclerosis, and target-lesion revascularization	Strut coverage: 95.0% (88.7–98.5) vs 91.4% (88.8–98.5); P=0.692. Neointimal thickness: 0.2 mm in both groups; P=0.471. Maximal obstruction: 15.5% vs 14.7%; P=0.801. Target-lesion revascularization: 0 vs three events; P=0.224.	Exenatide did not significantly improve early stent endothelialization or neointimal response. This is the most directly relevant mechanistic PCI report, but its small sample and short follow-up substantially limit precision.
Krychtiuk et al., 2024, Harmony Outcomes [15]	Post hoc coronary-outcome analysis of the parent multicenter, double-blind, placebo-controlled Harmony Outcomes randomized trial; 9,463 adults with T2D and established ASCVD	Albiglutide vs placebo, alongside standard care	Albiglutide 30 or 50 mg subcutaneously once weekly; median follow-up, 1.6 years	First and recurrent MI; type 1 and type 2 MI; STEMI/NSTEMI; other ischemic outcomes, including non-elective revascularization	Overall, 421 participants (4.5%) experienced at least one MI. Albiglutide reduced first MI: HR 0.75 (95% CI 0.62–0.91); total MI: HR 0.75 (95% CI 0.61–0.93); type 1 MI: HR 0.73 (95% CI 0.57–0.92); and type 2 MI: HR 0.65 (95% CI 0.46–0.92). Revascularization-related outcomes were not significantly reduced.	Albiglutide reduced first and recurrent MI across mechanistically different MI types. The subtype and recurrent-event analyses were exploratory, and a significant revascularization benefit was not demonstrated.
Barbery et al., 2021, EXSCEL [16]	Post hoc analysis of the parent randomized, placebo-controlled EXSCEL cardiovascular-outcome trial; 14,736 adults with T2D and complete follow-up data	Once-weekly exenatide vs placebo, alongside usual care	Extended-release exenatide 2 mg subcutaneously once weekly; median follow-up, 3.3 years (IQR 2.3–4.4)	Composite of hospitalization for ACS or coronary revascularization	The composite occurred in 1,642 participants (11.1%). Exenatide had no effect: HR 1.00 (95% CI 0.91–1.10). Established cardiovascular disease predicted higher risk: HR 3.24 (95% CI 2.78–3.78), as did previous MI: HR 1.54 (95% CI 1.39–1.71).	Exenatide did not reduce ACS hospitalization or coronary revascularization. The post hoc composite analysis did not specifically assess restenosis, target-lesion revascularization, stent healing, or treatment-initiation timing.

Risk-of-Bias Assessment

Overall RoB 2 judgments were derived from the five domain-level assessments for the results evaluated, rather than by calculating a numerical score. An overall low-risk judgment required all five domains to be rated low risk. An overall judgment of some concerns applied when at least one domain raised concerns, but no domain was rated high risk, and the combined concerns did not substantially lower confidence in the result. A high-risk judgment applied when at least one domain was rated high risk or concerns across multiple domains collectively substantially lowered confidence in the result [9].

FLOW (Evaluate Renal Function With Semaglutide Once Weekly) was judged low risk because all five domains were rated low risk [13]. For GRADE (Glycemia Reduction Approaches in Type 2 Diabetes: A Comparative Effectiveness Study), the overall judgment of some concerns arose from D4, reflecting the potential influence of open-label treatment on physician-dependent outcomes [11]. For AMPLITUDE-O (Effect of Efpeglenatide on Cardiovascular Outcomes), Harmony Outcomes, and EXSCEL (Exenatide Study of Cardiovascular Event Lowering), the overall judgments were driven by D5 concerns regarding selection of the reported result [12,15,16]. STOP (Semaglutide Treatment Effect on Coronary Atherosclerosis Progression) raised concerns in D1, D3, and D5 [10], while REBUILD (Effects of Exenatide on Coronary Stent Endothelialization in Subjects With Type 2 Diabetes) raised concerns in D1, D2, and D3 [14]. For these two reports, the combined concerns were judged insufficient to warrant an overall high-risk rating, although they limited confidence in the findings. The report-specific reasons supporting these assessments are presented in Table 2.

**Table 2:** Risk-of-bias assessment of the seven included reports from randomized trials, including primary reports and prespecified and post hoc analyses, using the Cochrane Risk of Bias 2 tool. ACS, acute coronary syndrome; AMPLITUDE-O, Effect of Efpeglenatide on Cardiovascular Outcomes; CKD, chronic kidney disease; CT, computed tomography; D1, bias arising from the randomization process; D2, bias due to deviations from intended interventions; D3, bias due to missing outcome data; D4, bias in the measurement of the outcome; D5, bias in the selection of the reported result; EAT, epicardial adipose tissue; EXSCEL, Exenatide Study of Cardiovascular Event Lowering; FLOW, Evaluate Renal Function With Semaglutide Once Weekly; GRADE, Glycemia Reduction Approaches in Type 2 Diabetes: A Comparative Effectiveness Study; MI, myocardial infarction; OCT, optical coherence tomography; RCT, randomized controlled trial; REBUILD, Effects of Exenatide on Coronary Stent Endothelialization in Subjects With Type 2 Diabetes study; RoB 2, Cochrane Risk of Bias 2 tool; STOP, Semaglutide Treatment Effect on Coronary Atherosclerosis Progression. Cochrane Risk of Bias 2 tool [9].

Study/trial	Tool applied	D1: Randomization process	D2: Deviations from intended interventions	D3: Missing outcome data	D4: Outcome measurement	D5: Selection of reported result	Overall risk	Basis for judgment
Manubolu et al., 2024 STOP [10]	Cochrane RoB 2	Some concerns	Low	Some concerns	Low	Some concerns	Some concerns	Double-blind, placebo-controlled design and objective CT measurements reduced performance and measurement bias. However, the brief report provided limited information about allocation concealment, completeness of serial imaging and prespecification of the EAT analysis.
Green et al., 2024 GRADE [11]	Cochrane RoB 2	Low	Low	Low	Some concerns	Low	Some concerns	Randomization, follow-up and prespecified analyses were generally robust. The open-label design could have influenced physician-dependent outcomes such as hospitalization and coronary revascularization, although cardiovascular events were centrally adjudicated and analyses followed randomized allocation.
Gerstein et al., 2023 AMPLITUDE-O [12]	Cochrane RoB 2	Low	Low	Low	Low	Some concerns	Some concerns	Randomized, double-blind, placebo-controlled design with objective, adjudicated cardiovascular outcomes. The dose-response analysis involved multiple secondary comparisons and trend analyses, creating some concern regarding selective emphasis and multiplicity.
Mahaffey et al., 2025 FLOW [13]	Cochrane RoB 2	Low	Low	Low	Low	Low	Low	Central randomization, double-blinding, placebo control, high follow-up completeness and blinded cardiovascular-event adjudication were used. The cardiovascular and CKD-severity analyses were prespecified, with no important evidence of selective reporting.
Santos-Pardo et al., 2023 REBUILD [14]	Cochrane RoB 2	Some concerns	Some concerns	Some concerns	Low	Low	Some concerns	Randomization procedures and allocation concealment were not described in sufficient detail. The study was open-label and only 33 of 38 randomized participants completed OCT follow-up. Nevertheless, OCT outcomes were prespecified, objectively measured and analyzed by a blinded independent core laboratory.
Krychtiuk et al., 2024 Harmony Outcomes [15]	Cochrane RoB 2 for a post-hoc randomized-trial analysis	Low	Low	Low	Low	Some concerns	Some concerns	The parent trial was a large double-blind RCT with blinded adjudication and nearly complete event ascertainment. However, MI subtypes, recurrent events and several ischemic outcomes were exploratory post-hoc analyses with multiple comparisons.
Barbery et al., 2021 EXSCEL [16]	Cochrane RoB 2 for a post-hoc randomized-trial analysis	Low	Low	Low	Low	Some concerns	Some concerns	The parent trial had randomized, double-blind treatment allocation, balanced discontinuation and near-complete follow-up. The ACS/revascularization composite was analyzed post hoc using univariate models, and the report did not fully address multiplicity or the potential data-driven selection of this composite outcome.

Discussion

Principal Findings and Overall Evidence Pattern

The available evidence suggests that GLP-1 RAs provide their clearest coronary benefits through reductions in major MACE and MI, rather than through consistent effects on revascularization or post-PCI vascular healing. In the post hoc Harmony Outcomes analysis, Krychtiuk et al. [15] reported lower risks of first, recurrent, type 1, and type 2 MI with albiglutide, whereas the prespecified dose-response analysis of AMPLITUDE-O by Gerstein et al. [12] demonstrated graded cardiovascular effect estimates favoring higher-dose efpeglenatide. The primary STOP trial report by Manubolu et al. demonstrated favorable changes in epicardial adipose tissue with semaglutide, while the prespecified cardiovascular analysis of FLOW by Mahaffey et al. reported a reduction in the composite of cardiovascular death, MI, or stroke among patients with diabetic kidney disease [10,13]. By contrast, the post hoc EXSCEL analysis by Barbery et al. [16] found no reduction in hospitalization for acute coronary syndrome (ACS) or coronary revascularization with exenatide, and the primary REBUILD trial report by Santos-Pardo et al. [14] found no significant improvement in early stent coverage or neointimal response following PCI. Collectively, these findings support an outcome-specific pattern of coronary protection rather than a uniform effect across all manifestations of coronary disease.

Surrogate Coronary Measures and Clinical Event Outcomes

The included reports evaluated distinct outcomes that should be interpreted separately. In the STOP trial, Manubolu et al. reported favorable changes in epicardial adipose tissue volume and density with semaglutide [10]. These are surrogate imaging measures and do not directly demonstrate reduced coronary inflammation, plaque stabilization, slower atherosclerotic progression, or prevention of MI. In contrast, the post hoc Harmony Outcomes analysis by Krychtiuk et al. reported reductions in first and recurrent MI, providing evidence concerning patient-important clinical outcomes, although the exploratory nature of these analyses warrants caution [15]. Neither report establishes that changes in epicardial adipose tissue mediate reductions in clinical events. Plaque stabilization, reduced vascular inflammation, improved glycemic and renal status, weight loss, and altered thrombotic susceptibility are biologically plausible explanations, but their individual contributions were not established by the included reports. Similarly, OCT-derived stent coverage and neointimal measures in REBUILD represent surrogate vascular-healing outcomes; its findings do not establish effects on clinical events such as stent thrombosis, MI, or repeat revascularization [14]. Thus, surrogate imaging findings should be considered hypothesis-generating and distinguished from demonstrated clinical outcome effects.

Interpreting Differences Between MI and Revascularization Findings

MI and coronary revascularization are patient-important clinical outcomes, but they reflect partly distinct processes. Revascularization is also a physician-mediated outcome influenced by symptom burden, evidence of ischemia, anatomic disease severity, procedural feasibility, comorbidities, patient preferences, and clinical urgency [17]. The geographic differences reported in the post hoc EXSCEL analysis by Barbery et al. illustrate variation in revascularization rates across health care settings, but do not establish the causes of that variation [16]. The neutral ACS/revascularization composite in EXSCEL and the reduction in MI in Harmony Outcomes arose from different trials involving different agents, populations, and outcome definitions [15,16]. These findings therefore cannot establish a consistent separation between MI prevention and revascularization effects across the GLP-1 RA class. One possible explanation is that these therapies influence plaque vulnerability or systemic cardiometabolic risk more readily than established coronary obstruction. However, this remains a biologically plausible hypothesis rather than a mechanism demonstrated by the included evidence.

Post-PCI Healing Represents a Distinct Therapeutic Question

Cardiovascular benefits observed in broad outcome trials cannot be assumed to translate directly into improved vascular healing after PCI. Native-vessel myocardial infarction is commonly related to plaque disruption and thrombosis, whereas post-PCI outcomes additionally depend on endothelial recovery, neointimal proliferation, stent expansion and apposition, platelet activity, local vascular inflammation, stent platform, antiplatelet therapy, and glycemic control. In the primary report of the REBUILD randomized trial, Santos-Pardo et al. addressed this question directly but randomized only 38 participants, obtained follow-up OCT imaging in 33, and evaluated vascular response after 12 weeks [14]. The high degree of strut coverage in both groups may also have created a ceiling effect that limited the ability to detect an incremental benefit from exenatide. Moreover, the trial was not adequately powered to assess relatively infrequent clinical outcomes such as stent thrombosis, restenosis, or target-lesion revascularization. OCT-derived strut coverage is a surrogate measure of vascular healing rather than a direct measure of clinical benefit [18]. These findings should therefore not be interpreted as evidence that GLP-1 receptor agonists have no post-PCI vascular effect, but rather that an improvement in early stent-healing measures was not demonstrated in this small trial [14].

Treatment Intensity, Agent Heterogeneity, and Patient Phenotype

The magnitude of coronary benefit may depend on biological exposure, drug-specific properties, and the clinical phenotype of the treated population. In the prespecified dose-response analysis of AMPLITUDE-O, Gerstein et al. reported more favorable cardiovascular effect estimates with 6 mg than with 4 mg of efpeglenatide [12]. Although tests for dose-response trend were statistically significant, the individual 4-mg comparisons for MACE and the expanded cardiovascular composite were not, and comparable dose-ranging evidence is unavailable for other agents. The findings therefore suggest, but do not establish, a dose-response relationship. Albiglutide, semaglutide, liraglutide, efpeglenatide, and exenatide also differ in molecular structure, pharmacokinetics, receptor exposure, and metabolic effects and should not automatically be regarded as interchangeable for coronary outcomes. Population characteristics may be equally important. Favorable estimates were generally more apparent in patients with established ASCVD, CKD, or high cardiovascular risk, whereas the prespecified cardiovascular-outcome analysis of GRADE by Green et al. provided more modest evidence in a comparatively lower-risk population, and the post hoc EXSCEL analysis by Barbery et al. found no effect of exenatide on ACS hospitalization or coronary revascularization [11,16]. These differences may reflect variation in baseline risk, follow-up, outcome definitions, background therapy, and statistical power rather than a definitive hierarchy among individual GLP-1 receptor agonists.

Timing of Initiation as a Missing Effect Modifier

Treatment timing may represent an important but largely unexamined determinant of coronary response to GLP-1 receptor agonists. Initiation before PCI could modify systemic inflammation, platelet activity, glycemic status, and metabolic stress at the time of vascular injury, whereas immediate post-procedural treatment might influence early endothelial recovery and local inflammatory signaling [2,3]. Continued therapy during the subsequent months could plausibly affect neointimal proliferation, restenosis, and maturation of stent coverage, while longer exposure may act predominantly through modification of native-vessel atherosclerosis and prevention of recurrent ischemic events. The 12-week intervention evaluated in the primary REBUILD trial report by Santos-Pardo et al. addressed only one part of this temporal continuum and did not compare different initiation strategies [14]. None of the parent randomized trials represented by the included reports directly compared pre-PCI, immediate post-PCI, and delayed initiation. Timing should therefore be regarded as a biologically plausible but unproven effect modifier and an important priority for future investigation.

Evidence Certainty and Interpretive Limitations

Interpretation of this evidence is constrained by substantial variation across the included reports and their parent trials in patient risk, GLP-1 receptor agonist, dose, comparator, treatment duration, and outcome definition. Coronary outcomes were frequently presented as secondary, composite, pooled, or post hoc analyses, and revascularization was seldom classified as target-lesion, target-vessel, urgent, or elective. Only the primary REBUILD trial report evaluated post-PCI vascular healing using direct intracoronary imaging, but its small sample, incomplete OCT follow-up, and short observation period limited precision [14]. None of the parent randomized trials was designed to determine whether treatment timing modified coronary response, while several favorable findings arose from prespecified dose-trend analyses or exploratory subtype, recurrent-event, and pooled-treatment analyses. The risk-of-bias assessment consequently identified some concerns in most included reports, principally because of post hoc analyses, incomplete methodological reporting, open-label treatment, missing imaging follow-up, and possible selective emphasis among multiple outcomes. Because only seven clinically heterogeneous reports were included and no quantitative meta-analysis was performed, formal assessment of publication bias or small-study effects using funnel plots or statistical tests for asymmetry was not appropriate. Publication bias, small-study effects, and the preferential publication of favorable secondary or post hoc findings therefore cannot be excluded. Furthermore, although the review methods were specified before screening commenced, the protocol was not prospectively registered or made publicly available, limiting independent verification that all methodological decisions were established a priori. Cardiovascular outcome trials also cannot distinguish direct vascular effects from benefits mediated through weight loss, glycemic improvement, renal protection, or broader cardiometabolic changes. Because the included outcomes represent distinct biological constructs rather than interchangeable measures of coronary benefit, statistical pooling would have produced a summary estimate of uncertain clinical meaning [1,4].

Clinical Implications and Future Research Priorities

GLP-1 receptor agonists with established cardiovascular benefit remain appropriate for patients with T2D and relevant cardiometabolic indications, particularly those with ASCVD or CKD [19], but current evidence does not justify their use specifically to accelerate stent healing or prevent restenosis. Future research could use a multicenter randomized design enrolling adults with T2D undergoing contemporary drug-eluting stent implantation, with stratification according to prior GLP-1 receptor agonist exposure and direct comparison of pre-PCI, immediate post-PCI, and delayed initiation. Serial OCT could evaluate stent coverage and neointimal response, while coronary CT could assess native-vessel progression. Clinical follow-up of at least 12 to 24 months should separately capture spontaneous MI, target-lesion revascularization, target-vessel events, stent thrombosis, and elective versus urgent procedures. Concurrent measurement of body weight, HbA1c, lipids, and renal markers could support analyses of potential metabolic and renal mediation, as explored in LEADER (Liraglutide Effect and Action in Diabetes: Evaluation of Cardiovascular Outcome Results) [20]. Additional assessment of platelet activity and inflammatory biomarkers could help investigate proposed vascular mechanisms. Overall, the included reports provide more evidence for reductions in MI and broader cardiovascular events than for improved revascularization or post-stent outcomes, although their heterogeneity limits direct comparisons and the latter outcomes remain insufficiently investigated rather than conclusively unaffected.

## Conclusions

Glucagon-like peptide-1 receptor agonists (GLP-1 RAs) appear to provide clinically meaningful coronary protection in adults with type 2 diabetes mellitus, with the strongest evidence supporting reductions in myocardial infarction and broader ischemic cardiovascular events. Their effects on coronary revascularization, restenosis, and post-percutaneous coronary intervention vascular healing remain uncertain, reflecting limited and heterogeneous evidence rather than a demonstrated absence of benefit. The observed separation between fewer ischemic events and largely neutral revascularization outcomes suggests that these therapies may influence plaque vulnerability and systemic cardiometabolic risk more readily than fixed coronary obstruction or the local vascular response to stent implantation. Overall, the coronary benefits of GLP-1 RAs should be regarded as outcome-specific and potentially dependent on the agent, dose, patient phenotype, and treatment timing rather than as a uniform class effect across the coronary disease continuum. Future trials should therefore move beyond composite cardiovascular outcomes and directly determine which patients, treatment windows, and vascular outcomes are most responsive to therapy.

## Appendices

Appendix A

**Table 3:** Summary of database search terms, Boolean structure, and search dates.

Database/platform	Search fields	Search terms and Boolean structure	Publication eligibility and final search
MEDLINE via PubMed	Title/abstract; MeSH terms where used	Population: "type 2 diabetes" OR "T2DM" AND Intervention: "GLP-1 receptor agonist" OR "semaglutide" OR "liraglutide" OR "exenatide" OR "albiglutide" OR "efpeglenatide" AND Outcomes: "myocardial infarction" OR "acute coronary syndrome" OR "coronary revascularization" OR "percutaneous coronary intervention" OR "PCI" OR "stent" OR "stent healing" OR "endothelialization" OR "in-stent restenosis" OR "restenosis" OR "target-lesion revascularization" OR "coronary plaque" OR "atherosclerosis progression" OR "epicardial adipose tissue" AND Randomized evidence: "randomized" OR "placebo" OR "clinical trial" OR "secondary analysis" OR "post hoc analysis"	Initial eligibility: January 1, 2020–December 31, 2025. Updated eligibility: through August 31, 2026. Final search: August 31, 2026.
Scopus	Article title, abstract, and keywords (TITLE-ABS-KEY)	The population, intervention, outcome, and randomized-evidence terms listed above, adapted to TITLE-ABS-KEY. Synonyms within each concept were grouped using OR; principal concepts were connected using AND.	Initial eligibility: January 1, 2020–December 31, 2025. Updated eligibility: through August 31, 2026. Final search: August 31, 2026.
Web of Science Core Collection	Topic (TS): titles, abstracts, author keywords, and Keywords Plus	The population, intervention, outcome, and randomized-evidence terms listed above, adapted to TS. Synonyms within each concept were grouped using OR; principal concepts were connected using AND.	Initial eligibility: January 1, 2020–December 31, 2025. Updated eligibility: through August 31, 2026. Final search: August 31, 2026.
Cochrane CENTRAL	Title, abstract, and keywords; controlled vocabulary where used	The population, intervention, outcome, and randomized-evidence terms listed above, adapted to the Cochrane Library interface and restricted to CENTRAL. Synonyms were grouped using OR; principal concepts were connected using AND.	Initial eligibility: January 1, 2020–December 31, 2025. Updated eligibility: through August 31, 2026. Final search: August 31, 2026.
